# Flow Patterns of Viscoelastic Fracture Fluids in Porous Media: Influence of Pore-Throat Structures

**DOI:** 10.3390/polym11081291

**Published:** 2019-08-02

**Authors:** Xiaoxi Yu, Yuan Li, Yuquan Liu, Yuping Yang, Yining Wu

**Affiliations:** 1 School of Chemical Engineering, China University of Petroleum (East China), Qingdao 266580, China; 2 School of Petroleum Engineering, China University of Petroleum (East China), Qingdao 266580, China; 3 CNPC Engineering Technology R&D Company Limited, Beijing 102206, China

**Keywords:** viscoelastic surfactant fluid, hydrolyzed polyacryamide solution, pore-throat structure, flow patterns

## Abstract

Viscoelastic surfactant (VES) fluid and hydrolyzed polyacryamide (HPAM) solution are two of the most common fracturing fluids used in the hydraulic fracturing development of unconventional reservoirs. The filtration of fracturing fluids in porous media is mainly determined by the flow patterns in pore-throat structures. In this paper, three different microdevices analogue of porous media allow access to a large range of Deborah number (De) and concomitantly low Reynolds number (Re). Continuous pore-throat structures were applied to study the feedback effect of downstream structure on upstream flow of VES fluid and HPAM solution with Deborah (De) number from 1.11 to 146.4. In the infinite straight channel, flow patterns between VES fluids and HPAM solution were similar. However, as pore length shortened to 800 μm, flow field of VES fluid exhibited the triangle shape with double-peaks velocity patterns. The flow field of HPAM solution presented stable and centralized streamlines when *Re* was larger than 4.29 × 10^−2^. Additionally, when the pore length was further shortened to 400 μm, double-peaks velocity patterns were vanished for VES fluid and the stable convergent flow characteristic of HPAM solution was observed with all flow rates.

## 1. Introduction

Conventional oil and gas resources are steadily diminishing worldwide [1,2]. Most of the remaining oil and gas resources are unconventional oil and gas reservoirs, such as coalbed methane, shale gas, and tight oil and gas reservoirs. [3,4]. Therefore, the efficient and economic development of unconventional reservoirs has become an important research area [5,6]. Hydraulic fracturing technology is one of the most effective techniques to increase the productivity of unconventional reservoirs [7,8,9]. In this technique, a particular viscoelastic fluid injected into the formation is referred as a fracturing fluid. The type and performance of fracturing fluids is the key to successful application of fracturing processes. Polymer fracturing fluid (HPAM solution) and viscoelastic surfactant (VES) fluids are the two most representative fracturing fluid systems in low permeability reservoirs [10,11,12,13].

Filtration loss of fracturing fluid is one of the most important indexes for evaluating the performance of fracturing fluids. [14]. Compared with polymer fluids, the VES fluids have a much higher filtration loss to reservoir matrix. Excessive filtration loss increases development costs and reduces economic benefits [15,16], which is a serious problem for further application. It was found that the flow characteristics of the fracturing fluid in the porous media affect the amount of filtration loss. However, research in this field is still insufficient. Therefore, further experimental studies are needed to clarify the flow characteristics of fracturing fluids in pore throat structures [17,18].

VES fluids are composed of low molecular weight surfactants. The surfactants are weakly bound by non-covalent bonds that repeatedly undergo scission and recombination in dynamic equilibrium. As the surfactant concentration increasing, the spherical micelles will transform into giant wormlike micelles and fluid viscosity will increase [19,20]. What’s more, the viscoelastic properties can recover from micellar degradation in strong shearing and stretching deformation [21,22,23,24,25,26,27].

In recent years, microfluids have become a common method for dealing with fluids at micro-length scale, especially for generating and operating complex fluids with controllable size and customized structure [28,29]. Furthermore, it has been developed as microfluidic rheometers or tools to study new flow characteristics, providing a powerful and efficient way to simulate the flow patterns of the VES fluids in geometric structures similar to porous media [30].

However, previous study of the microfluids are focusing on single abrupt contraction–expansion geometry, while the research on the flow characteristics in the continuous pore-throat structure are insufficient and unclear [31]. In this work, we present the flow patterns of the VES fluids and the HPAM solutions in two continuous pore-throat structures with different length to observe the deformations of the flow patterns. The evolution process of all solutions with increasing injection rates is captured and recorded by microparticle image velocimetry (μ-PIV) in the first place. The three-dimensional velocity fields and velocity magnitude maps are illustrated in detail to further reveal the feedback effect of downstream structure on upstream flowing.

## 2. Materials and Experimental Methods

### 2.1. Microchannel Design and Fabrication

**Figure 1:**
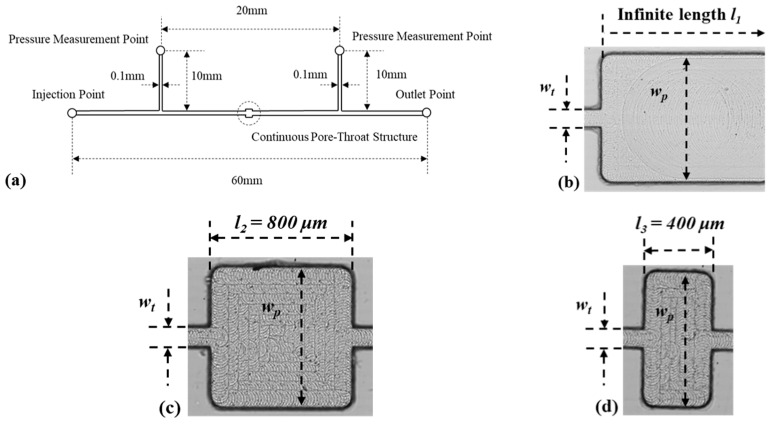
(**a**) Schematic diagram of the microfluidic device. (**b**) Pore-throat structure of device 1. (**c**) Pore-throat structure of device 2. (**d**) Pore-throat structure of device 3.

The microfluidic devices were fabricated on a plate (100 × 80 × 10 mm) of polymethyl methacrylate (PMMA) by precisely milling and sealed with another PMMA plate (100 × 80 × 3 mm). The layout of the device is displayed in Figure 1. All the microfluidic devices consisted of an injection point, an outlet point, a liner microchannel and a continuous pore-throat structure with two pressure taps located 10 mm away at upstream and downstream. The continuous pore-throat structure had a contraction–expansion structure at upstream and an expansion–contraction structure at downstream (Figure 1b). The solution at the inlet was connected to an independent syringe (10 mL, Hamilton, Germany) driven by a syringe pump (Harvard Apparatus, PHD2000, Plymouth Meeting, PA USA) to obtain accurate flow rates ranging from 0.5 mL/h to 12 mL/h.

The microfluidic devices with three different continuous pore-throat structures were fabricated as mentioned to investigate the effect of flow length. Pore width w_p_ = 800 μm, throat width of w_t_ = 100 μm as well as the pore length *l* are illustrated in Figure 1a.

### 2.2. Fluid Rheological Characterizations and Dimensionless Number

Two kinds of viscoelasticity solutions were prepared in this study, and a Newtonian fluid was used as the contrast. The viscoelasticity fluids were 0.2% hydrolyzed polyacrylamide (HPAM) and water solutions with HPAM molecular weights of 9.6 × 10^6^ g/mol. The VES fluid composed of 25 mMol/L cetyltrimethylammonium bromide (CTAB) and 12 mMol/L sodium salicylate (NaSal). In contrast, the Newtonian fluid was 93% glycerol-water solution to match the approximate viscosity of 95 mPa s. All the solutions were mixed for at least 24 h under temperature 25 °C.

**Figure 2:**
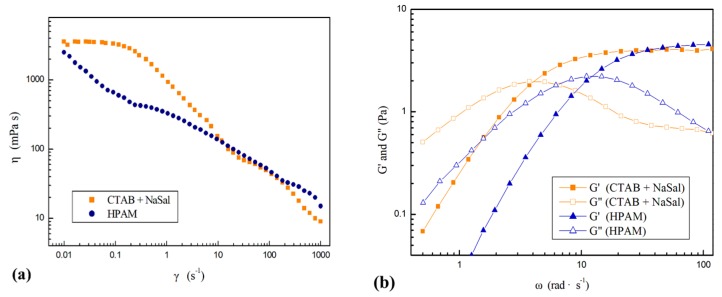
Viscosity *η* of the VES fluid and the HPAM solution as shear rates γ rising from 0.01 s^−1^ to 1000 s^−1^. and (**b**) Oscillatory measurement of the storage modulus G′ and the loss modulus G″.

HAAKE MARS 60 Rheometer were equipped with a cylinder rotor at 25 °C to test the rheological characteristics of all solutions. Before testing, samples were kept stable for 5 min to reach equilibrium. The oscillatory measurements were proceeded with shear rates rising from 0.01 s^−1^ to 1000 s^−1^. Due to the rheological characteristics of shear thinning, the viscosities (*η*) of both solutions were similar while shearing rates γ > 5 s^−1^ (shown in Figure 2). The relaxation times were 1.41 s for the VES fluid and 0.51 s for the HPAM solution. In contrast, the Newtonian fluid was 83% glycerol–water solution with a viscosity of 75 mPa s.

The dimensionless numbers were used in this investigation to quantitate the deformation of the macroscopic flow field observed in different pore-throat structures. The Reynolds number was defined by the average flow velocity $\overset{-}{V_{p}}$ in pore channel, in which $D_{h}=2w_{p}h/(w_{p}+h)$:

(1) $$Re=\frac{\rho \overset{-}{V_{p}}D_{h}}{\eta_{p}}=\frac{2\rho Q}{(w_{p}+h)\eta_{p}}$$

Deborah number (De) is the ratio of a solution’s relaxation time to residence time in the flow as:

(2) $$De=\frac{\lambda_{1}}{w_{p}lh/Q}=\frac{\lambda_{1}Q}{w_{p}lh}$$

where *Q* is the injection rate, *h* is the depth of the microchannels and *l* is the length of the pore-throat structures.

### 2.3. Flow Visualization

In order to obtain and analyze flow fields of contraction and expansion structures, micro-particle image velocimetry, μPIV (LaVision. Ltd) was used to perform quantitative measurements on the flow kinematics. For this purpose, mono-disperse fluorescent polystyrene microspheres, with 0.1 mL per 50 mL of bulk fluids, were blended into the fluids. A quite low magnification Zeiss Plan-Neofluar objective lens was used to capture the full width of the flow channel and a reasonable length of both upstream and downstream. Using a light sheet, formed by passing a double pulsed laser beam through an optical arrangement including cylindrical lenses, the fluorescent particles in the flow are illuminated twice within a small time separation between (dt). The images of particles displacement between the laser pulse were captured and recorded by the CCD (charge coupled device) camera. The whole velocity vectors were calculated by a conventional cross-correlation PIV algorithm.

## 3. Result and Discussion

### 3.1. Flow Characteristics in Infinite Length Pore-Throat Structure

**Figure 3:**
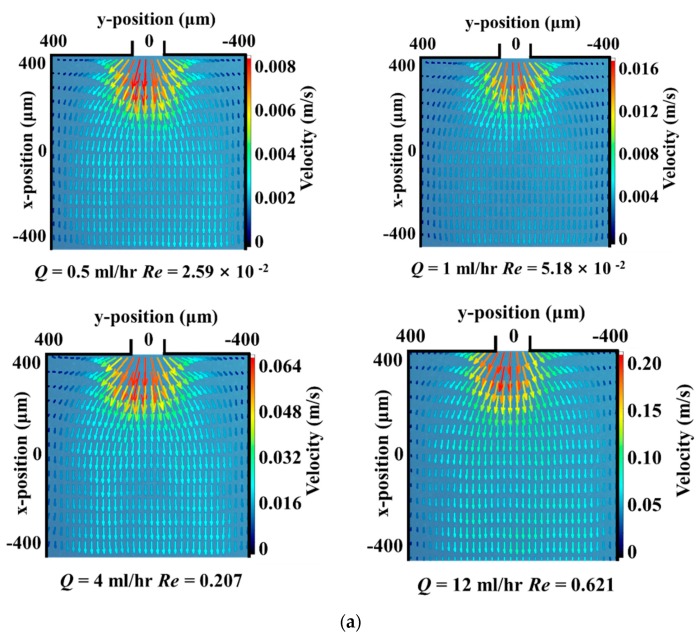

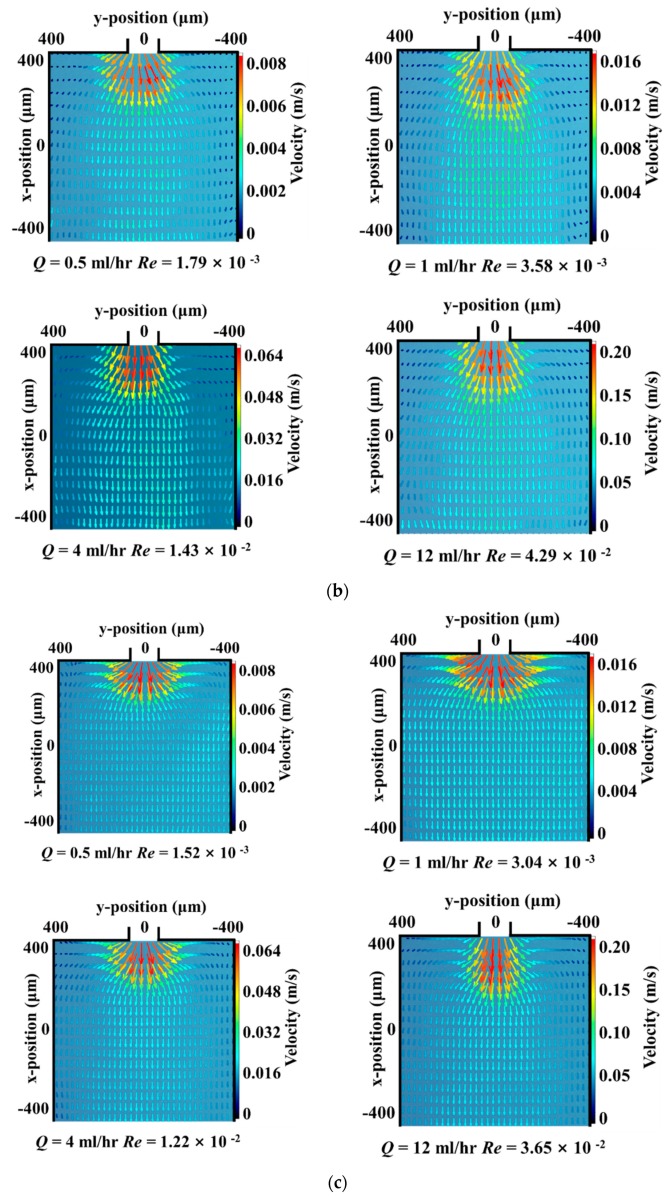
The development of flow patterns for the flow of (**a**) Newtonian fluid, (**b**) HPAM solution and (**c**) VES fluid in infinite length pore-throat structure, flow rates range from 0.5 mL/h to12 mL/h.

The flow characteristics of all solution were first examined in the microchannel with single pore-throat structure at upstream. Here, the downstream pore structure was treated as an infinite straight channel, which eliminates the impact of downstream structure on flow field in the pore. As displayed in Figure 3, the flow characteristics of all three solutions exhibited similar flow patterns with different Reynolds numbers (Re). After flowing through the entrance at upstream, the streamlines consisted of velocity components on both x-axis and y-axis directions. The velocities from the centerlines and the boundaries were different in the beginning. Afterwards, the velocity at centerlines gradually reduced until arriving at a relatively stable value. Finally, the streamlines tended to follow the stable linear flow expected behaviors, that is to say the velocity component was along the x-direction and barely changed. The difference of the divergent degree was visible among three solutions. The HPAM solution exhibited the most convergent patterns, while the VES fluid was observed as the most divergent patterns.

### 3.2. Flow Characteristic in Long Pore-Throat Structure

**Figure 4:**
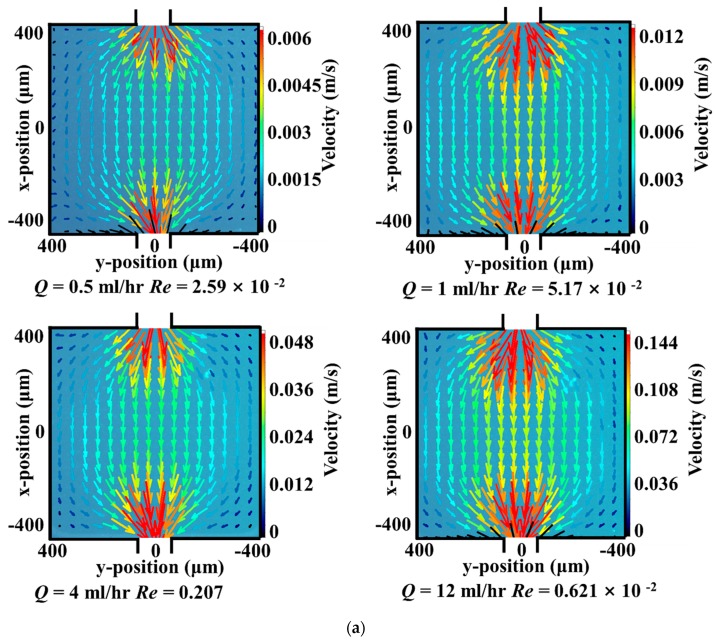

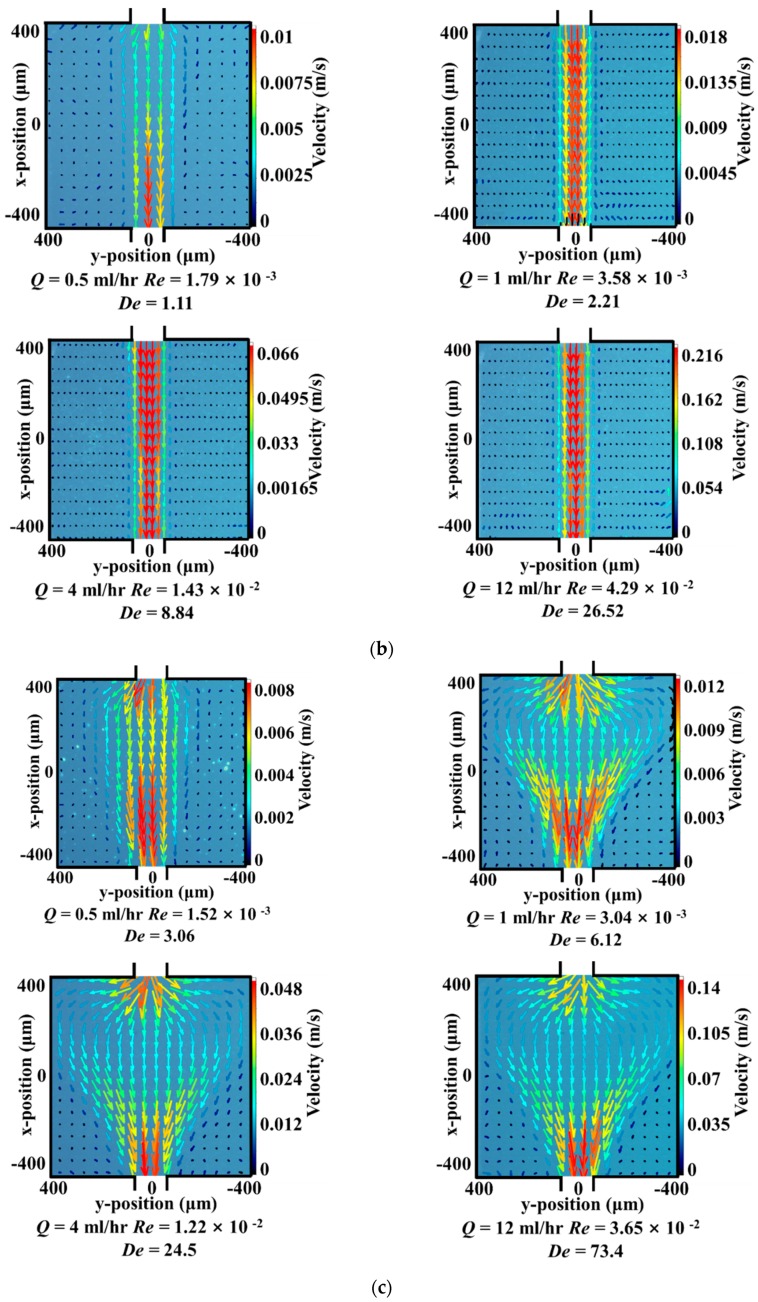
The development of flow patterns for the flow of (**a**) Newtonian fluid, (**b**) HPAM solution and (**c**) VES fluid in long pore-throat structure, flow rates range from 0.5 mL/h to12 mL/h.

The velocity fields of the Newtonian fluid in long continuous pore-throat structures were illustrated in Figure 4a. When Newtonian fluid flowed through the continuous pore-throat structure, the streamline underwent three stages including the divergent flow, the stable flow and the contractive flow. The maximum velocity was observed at the entrance due to the gathering of streamlines. After that, the streamlines performed to be divergent from the centerlines to boundaries. This flow stage was signed as the divergent flow. Then the streamlines tended to follow the stable linear flow expected behaviors, in other words, entered the stable flow stage. Afterwards, the streamlines got gathering from surrounding to centerlines causing the increase of velocity near the exit, which was classified as the contractive flow. It was found that all the velocity fields of the Newtonian fluid in continuous pore-throat at different flow rates (Re number increasing from 2.59 × 10^−2^ to 0.621) presented the same flow pattern. Namely, the rising of flow rate has little effect on the transformation of the flow patterns. In addition, the upstream and downstream velocity fields of microchannels were basically symmetrical.

When it came to the HPAM solution, at low Re number (Re = 1.79 × 10^−3^), the streamlines were still divergent to the boundaries at upstream. The streamlines tended to gather to the centerlines at downstream. However, unlike the flow pattern of Newtonian fluid, the gathering of streamlines was observed farther away from the exit of microchannel, about 250 μm before expansion–contraction structure. As the Re number rising to 4.29 × 10^−2^ (shown in Figure 4b), the flow of HPAM solution only experienced one stage that the flow patterns kept stable from the entrance to the exit, and there was no dispersion of streamlines from the centerlines to the boundaries. Additionally, the arrows of velocity in flow fields showed that there were only velocity components along the flow direction on the x-axis direction. The velocities at boundaries were quite small compared with the centerlines.

As shown in Figure 4c, the velocity fields of the VES fluid were measured with flow rates spanning 0.5 < *Q* < 12 mL/h. The flow patterns of the VES fluid was similar with the HPAM solution at low Re number rates (1.52 × 10^−3^), but transformed into new patterns as Re number larger than 3.04 × 10^−3^. The velocity fields of VES fluid at high flow rates were illustrated as triangle shape, which was distinct from the HPAM solution. When flow rates were higher than 2 mL/h, there was almost no velocity component on *y*-axis direction along the streamline of the HPAM solution. However, the streamlines of VES fluid still maintained the characteristics of Newtonian-like flow at upstream of the long continuous pore-throat structures. The streamlines of VES fluid were observed to have the contractive behavior far away from the expansion–contraction structure. The streamlines were accelerated to flow from the boundary to the exit at about 300 μm from the entrance, leading to the contractive behavior of flow patterns. In addition, the velocities of streamlines outside the triangle shape flow fields were close to zero. In those areas, flow rates were very minimal compared with the main flow areas.

### 3.3. Flow Characteristic in Short Pore-Throat Structure

**Figure 5:**
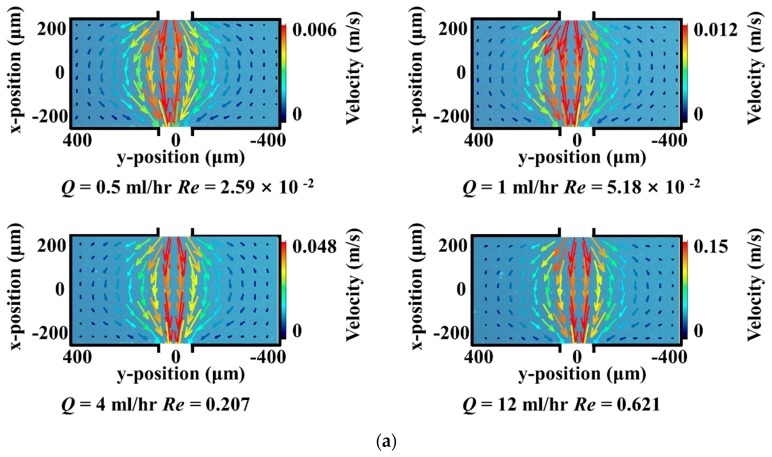

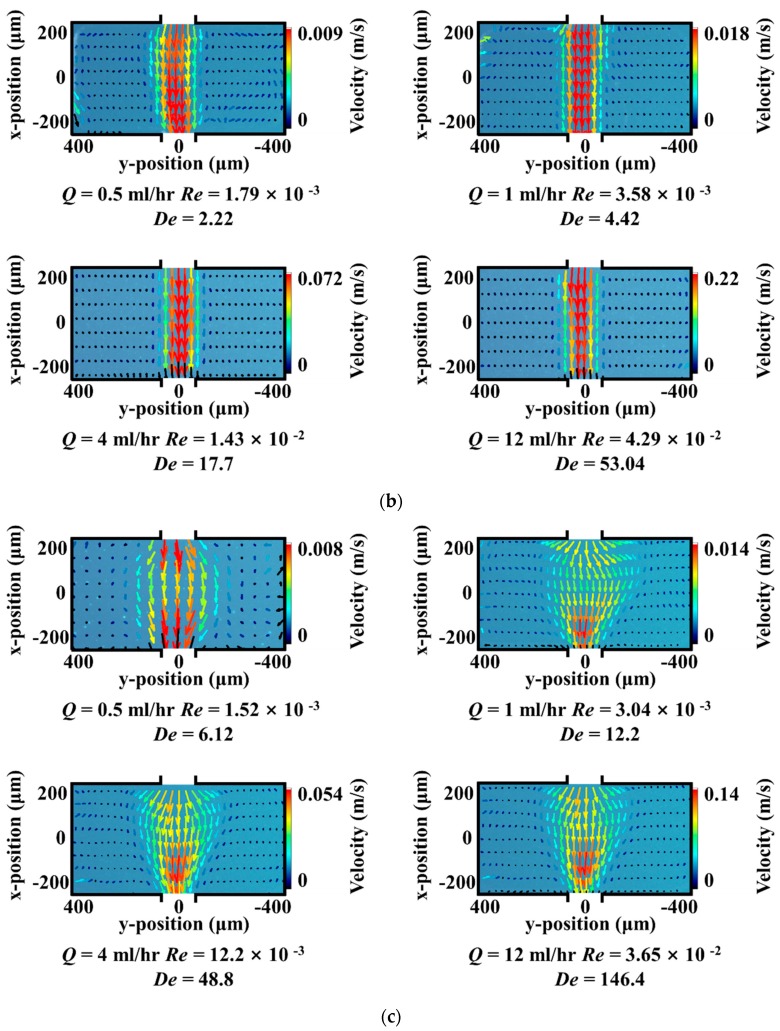
The development of flow patterns for the flow of (**a**) Newtonian fluid, (**b**) HPAM solution and (**c**) VES fluid in short pore-throat structure, flow rates range from 0.5 mL/h to12 mL/h.

The results of the Newtonian fluid, HPAM solution and VES fluids in short continuous pore-throat structure are shown in Figure 5. The velocity fields indicated that the flow patterns of Newtonian fluid were barely affected by the length of the pore-throat structure and flow rates. Similar with the flow characteristics from Figure 4a, the process of Newtonian flow was separated into the divergent flow, the stable flow and the contractive flow. Even though the length of the pore-throat structure were shortened to 400 μm.

As shown in Figure 5b, in the case of the HPAM solution, at low flow rates (Re = 1.79 × 10^−3^) there was an existence of the dispersion of streamlines observed in the microchannel at upstream. Compared with the μ-PIV velocity field in Figure 4b at Re = 1.79 × 10^−3^, the streamlines in short microchannel performed a more distinct contractive behavior. This phenomenon was generated by the influence of the length between the structure entrance and exit. According to the former study on the Newtonian fluid and HPAM solution in long continuous pore-throat structures, the streamlines would experience contractive flow stage before going through the exit at downstream. The elastic of HPAM solution was capable to result in the stronger gathering of streamlines. The distinct contractive behavior in Figure 5b was caused by the length of the microchannel. Before the streamlines were able to show the Newtonian-like divergent characteristic, streamlines have already been influenced by the contractive behavior at downstream. However, the flow patterns were similar with Figure 4b at higher flow rates (Re = 1.43 × 10^−2^ and 4.29 × 10^−2^).

The more distinct contractive behavior of streamlines was also observed as VES fluid flowing through short microchannel shown in Figure 5c. Flow patterns of VES fluid were more similar with Newtonian fluid compared with HPAM solution, particularly at Q = 0.5 mL/h. The streamlines were observed with divergent behavior at all flow rates increasing from 0.5 mL/h to 12 mL/h, while the flow patterns of HPAM solution were contraction flow at Q = 1 mL/h and 12 mL/h. However, there were obvious differences between the flow pattern of VES fluid in long and short pore-throat structure, with the streamlines performed as triangle shape velocity fields as flowing through the long microchannel. While the flow length was shortened to 400 μm, the arrows of velocity in short microchannel were mostly along the x-direction. This is different from the velocity components shown in Figure 4b. Because of lacking enough velocity components along the vertical flow direction, the divergent flows of VES fluid were not obvious compared with the triangle shape flow patterns visualized in the long microchannel.

As non-Newtonian fluids flowing through the continuous pore-throat structures, the flow patterns were influenced by the structure of upstream entrance, downstream exit and the flow length. There were divergent flows at upstream and contractive flows at down, due to the sudden change of width in pore-throat structures. However, the elasticity of both HPAM solution and VES fluid affected the streamlines distribution near the entrance and exit. With the influence of fluids elasticity, there was a process of streamlines to establish the flow patterns after flowing out or before entering the width changing structures of pore-throat. The length between the entrance and exit determined the process space to generate the divergent flow or contractive flow. In the short microchannel, the flow patterns at upstream were influenced by the downstream structure to come into being the insufficiency divergent flow.

### 3.4. Velocity Distribution in Continuous Pore-Throat Structure

**Figure 6:**
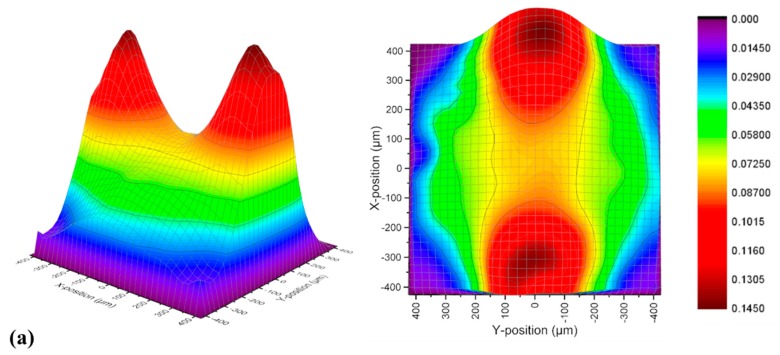

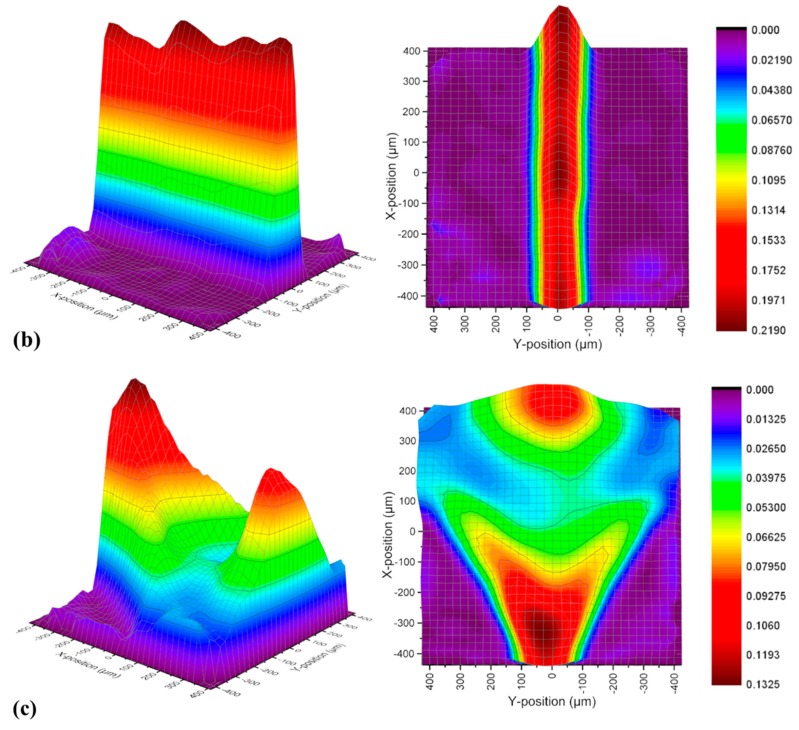
The 3D velocity fields of the (**a**) Newtonian fluid; (**b**) HPAM solution and (**c**) VES fluid in long continuous pore-throat structure at *Q* = 12 mL/h.

**Figure 7:**
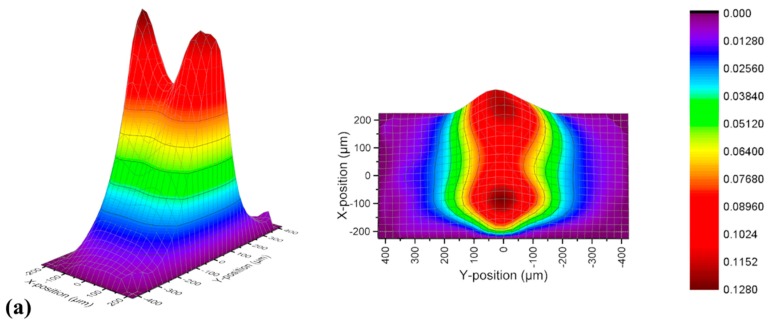

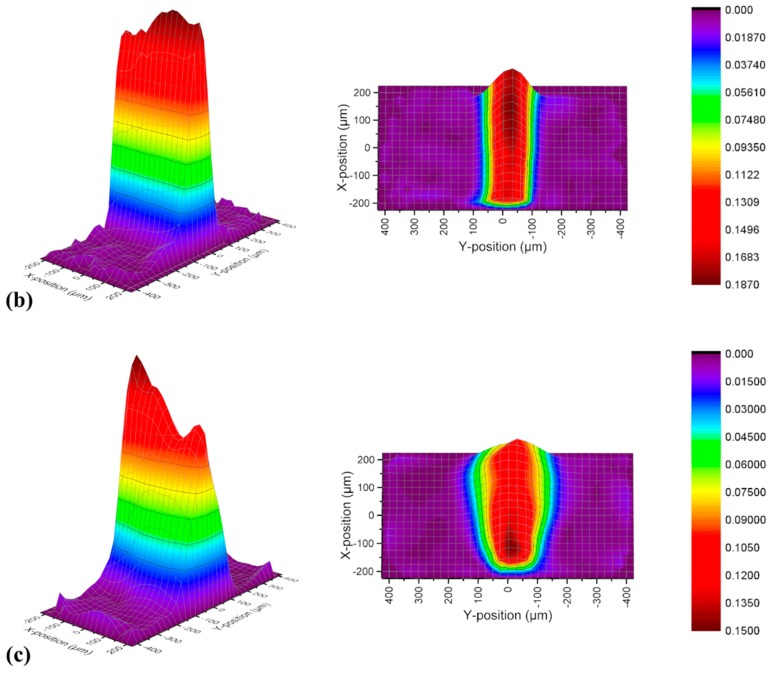
The 3D velocity fields of the (**a**) Newtonian fluid; (**b**) HPAM solution and (**c**) VES fluid in long continuous pore-throat structure at *Q* = 12 mL/h.

To quantify the flow pattern differences among those three solutions in both pore-throat structures, matrix tool was used to establish 3D velocity fields at *Q* = 12 mL/h. The heights of 3D-velocity fields represented the velocity at each point (located by x-position and y-position), which were marked with different colors as shown in Figure 6 and Figure 7.

The velocity contour in those 3D velocity fields primly explained the velocity distributions seized by μ-PIV equipment. The flow pattern of the Newtonian fluid was nearly symmetrical in Figure 6a with the ellipsoid shape, which was similar to the former study of Rodd [32]. The streamlines of the HPAM solution has shown a strong contractive behavior at high flow rates as the velocity component of each point was almost always along *x*-axis. The 3D velocity field of the HPAM solution was observed as a banded shape where most of the streamlines were gathering (shown in Figure 6b). Thus, other area outside the banded shape owned little velocity. At the upper part in Figure 6c, the velocity distribution of the VES fluid was similar with the Newtonian fluid that the streamlines diverged gently to each area (from x = 400 μm to x = 200 μm). After that, two high velocity isoline parts were generated at both sides causing the gathering of streamlines, accelerating the contractive stage of the VES fluid flow patterns.

**Figure 8:**
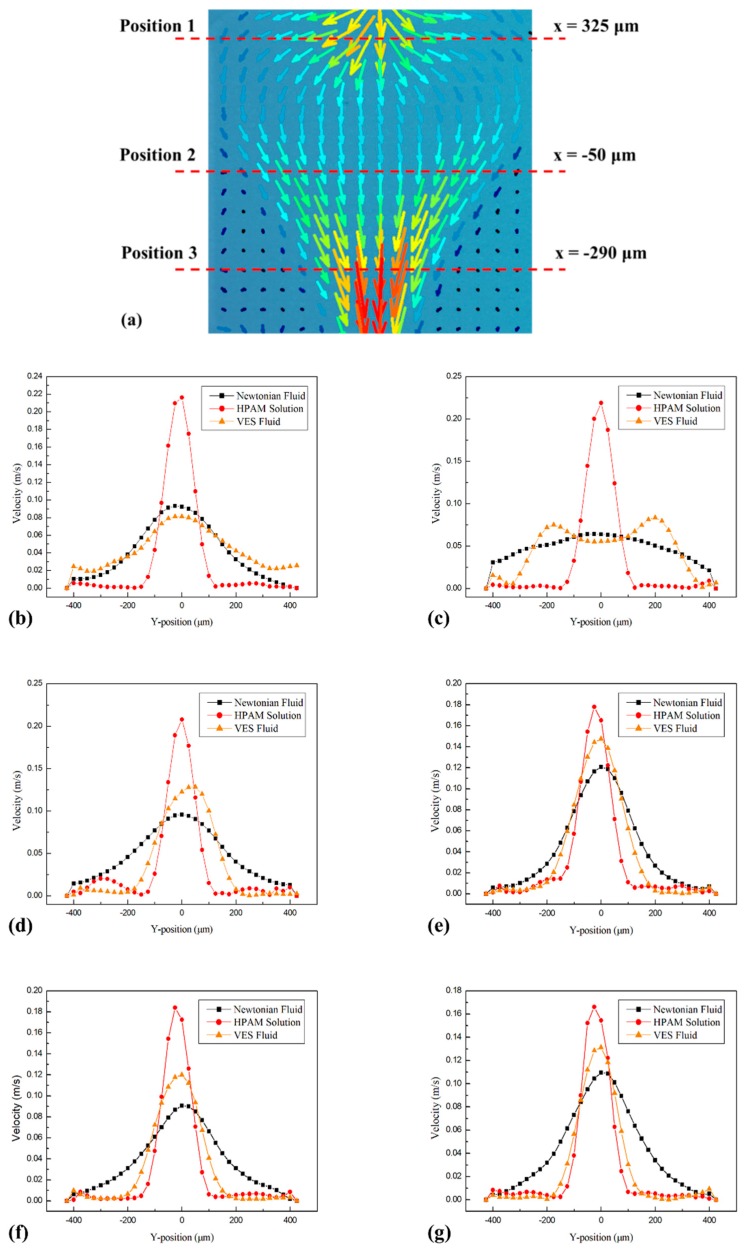
(**a**) The schematic of velocity line positions; Velocity magnitude maps of the Newtonian fluid, HPAM solution and VES fluid in long continuous pore-throat structure at (**b**) x = 325 μm; (**c**) x = −50 μm, (**d**) x = −290 μm and in short continuous pore-throat structure at (**e**) x = 150 μm, (**f**) x = 0 μm, (**g**) x = −75 μm.

Furthermore, the 3D velocity fields and velocity magnitude lines of all solutions flowing through short continuous pore-throat structure at *Q* = 12 mL/h are illustrated in Figure 8. All the data of the Newtonian fluid are used as the standard comparison. It can be seen that the length of pore channel has little effect on the flow characteristics of the Newtonian fluid and the HPAM solution.

However, the VES fluid presented a different flow pattern. As mentioned in Figure 4c, the behavior of VES fluid was divergent flow and similar with the Newtonian fluid at upstream. But it was more contractive than the Newtonian fluid, which was in contrast with the result in the long continuous pore-throat structure (Figure 6c). Comparing Figure 8b,e, there was no appearance of the velocity along the y-direction and ‘approximate inviscid flow area’ near the contraction–expansion structure in the short pore-throat structure. At the middle area of the structure, flow patterns developed into double-peaks velocity line in the long structure (Figure 8c), while kept a single-peak shape as well in Figure 8f. After flowing near the exit at downstream, they reappeared as a similar shape (Figure 8d,g). In general, the structure of downstream was able to affect the development of flow pattern upstream.

## 4. Conclusions

Viscoelastic fluids underwent shear and extensional flow when flowing in porous media as characterized by pore-throat structures. Because of the feedback effect, the pore length has significant influence on the distribution of velocity fields. In continuous pore-throat structures, the distance between the upstream and downstream structures determined the development degree of flow patterns, especially for VES fluid. For infinite length distance, the extensional deformations of both VES fluid and HPAM were negligible without the contraction at downstream. As the flow length shortened to 800 μm, there were double-peak velocity patterns obtained for VES fluid. However, this pattern vanished at a microchannel length of 400 μm. Through this work, we have clearer knowledge about the characteristics of the viscoelastic surfactant fracture fluid in porous media, which is beneficial for developing hydraulic fracturing technology.
